# Supplementary material for: Identification of photoperiod-induced specific miRNAs in the adrenal glands of Sunite sheep (Ovis aries)
Source: Front Vet Sci. 2022 Jul 22;9:888207. doi: 10.3389/fvets.2022.888207 (PMC9354845; doi:10.3389/fvets.2022.888207)
Supplement: Supplementary file 1 [file Data_Sheet_1.ZIP › Supplementary file/miRDeep2 file.zip!/miRDeep2 file/Know_Structure/SP42A1/oar-mir-154b.pdf]

miRBase precursor : oar-mir-154b
Total read count : 1052
oar-miR-154b-5p read count : 981
oar-miR-154b-3p read count : 66
remaining reads : 5

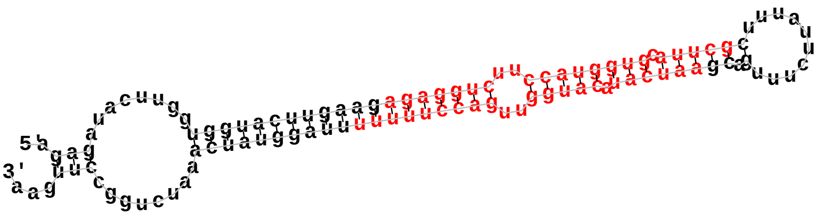

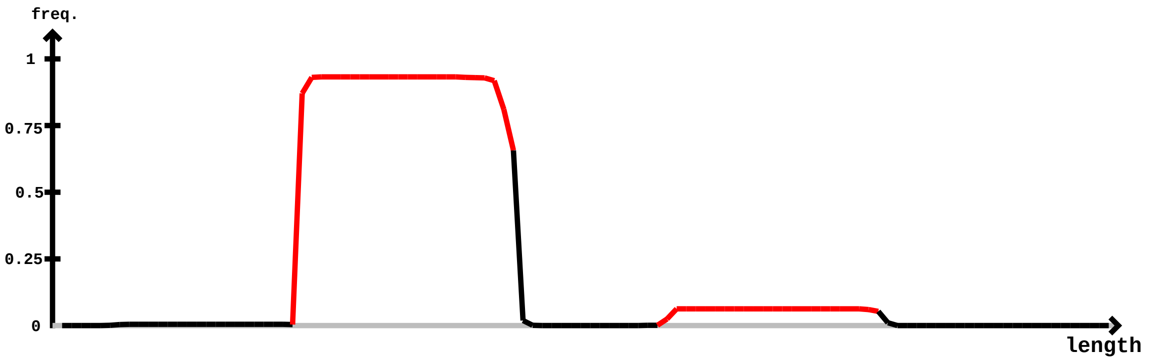


```
                                                                 oar-miR-154b-3p
                         oar-miR-154b-5p
5'- agagauacuuggugguacuugaagagaggucuuccauggugcauucgcuuuauucuuugacgaaucauacaugguugaccuuuuuuuagguaucaaaucuggccuugaa   -3'    exp
    .(((........((((((((((((((((((..((((((((.((((((..........).)))))))).))))).))))))))))))))))))........)))...     reads     mm     sample
    ....auacuuggugguacuugaa..........................................................................................     1         0      seq
    .....uacuuggugguacuugaag.........................................................................................     3         0      seq
    ......acuuggugguacuugaag.........................................................................................     1         0      seq
    ........................agaggucuuccaugguA....................................................................     1         1      seq
    ........................agaggucuuccauggug....................................................................     1         0      seq
    ........................agaggucuuccauggugc...................................................................     1         0      seq
    ........................agaggucuuccauggugca..................................................................     1         0      seq
    ........................agaggucuuccauggugcau.................................................................     11        0      seq
    ........................agaggucuuccauggugcauu................................................................     107       0      seq
    ........................agaggucuuccauggugcauuc...............................................................     154       0      seq
    ........................agaggucuuccauggugcauucg..............................................................     622       0      seq
    ........................agaggucuuccauggugcauucgc.............................................................     18        0      seq
    .........................gaggucuuccauggugcauu................................................................     6         0      seq
    .........................gaggucuuccauggugcauuc...............................................................     8         0      seq
    .........................gaggucuuccauggugcauucg..............................................................     48        0      seq
    .........................gaggucuuccauggugcauucgc.............................................................     1         0      seq
    ..........................aggucuuccauggugcauucg..............................................................     1         0      seq
    ..........................aggucuuccauggugcauucgcu............................................................     1         0      seq
    ............................................................cgaaucauacaugguugaccuuu..........................     1         0      seq
    ..............................................................aaucauacaugguugaccuuu..........................     2         0      seq
    ..............................................................aaucauacaugguugaccuuuu.........................     6         0      seq
    ..............................................................aaucauacaugguugaccuuuuu........................     15        0      seq
    ..............................................................aaucauacaugguugaccuuuuuu.......................     2         0      seq
    ...............................................................aucauacaugguugaccuuu..........................     1         0      seq
    ...............................................................aucauacaugguugaccuuuuu........................     30        0      seq
    ...............................................................aucauacaugguugaccuuuuuu.......................     9         0      seq
```
